# Resident eosinophils in patients with chronic obstructive pulmonary disease: a pilot study

**DOI:** 10.3389/fmed.2026.1814890

**Published:** 2026-05-25

**Authors:** Wei Sun, Sijie Liu, Ruonan Xu, Jiawei Jin, Chao Ren, Jing Wang

**Affiliations:** Department of Respiratory and Critical Care Medicine, Beijing Institute of Respiratory Medicine and Beijing Chao-Yang Hospital, Capital Medical University, Beijing, China

**Keywords:** chronic obstructive pulmonary disease, cytokine, eosinophil, prognosis, type 2 inflammation

## Abstract

**Background and objective:**

Eosinophilic inflammation is involved in approximately 20% of patients with chronic obstructive pulmonary disease (COPD). Research examining the role of eosinophils in the initiation and progression of COPD has yielded varying conclusions, potentially attributable to the different subtypes of eosinophils.

**Methods:**

In this prospective observational study from Jan 2025 and Jun 2025, we included 27 patients with stable COPD and 10 controls. Blood samples were collected for eosinophil isolation, and flow-cytometry analysis was used to determine the subtype of eosinophil: resident eosinophils (rEos: Siglec-8^+^CD62L^+^IL-3R^low^) or inflammatory eosinophils (iEos: Siglec-8^+^CD62L^low^IL-3R^hi^). We also recorded and analyzed baseline characteristics, pulmonary function test results, and several cytokines.

**Results:**

The absolute number and proportion of rEos was significantly lower in patients with stable COPD compared to healthy controls. The absolute number of rEos (cell/uL) serves as a biomarker for predicting COPD (AUC 0.856; 95% CI 0.736–0.975). A ratio below 1.955 (odds ratio 0.231; 95% CI 0.063–0.841; *p* = 0.026) may be linked to COPD pathophysiology.

**Conclusion:**

rEos levels were notably lower in stable COPD patients compared to healthy individuals and were associated with COPD presence. Future research should include larger, diverse groups in multicenter studies to confirm the role of rEos in COPD and conduct basic experiments to investigate the molecular and cellular pathways of eosinophilic phenotypes in COPD patients.

## Background

Chronic obstructive pulmonary disease (COPD) is a heterogeneous disease and the inflammation of the respiratory tract in patients with COPD has traditionally been attributed to the activity of lymphocytes and neutrophils (1). Nonetheless, increasing evidence indicates that approximately 20% of patients with stable COPD exhibit eosinophilic airway inflammation, as determined by sputum eosinophil counts (2). This eosinophilic inflammation is correlated with the occurrence of exacerbations (3) and demonstrates a responsiveness to corticosteroid treatment (4).

Studies investigating the role of eosinophils in the onset and progression of acute exacerbations of chronic obstructive pulmonary disease (AECOPD) have produced varying conclusions. Eosinophilia was linked to higher risks of 12-month COPD-related and all-cause readmissions, as well as a shorter time to the first COPD-related readmission (5). However, Greulich proved that high blood eosinophil counts (BEC) are associated with a shorter length of hospital stay in exacerbated COPD patients (6). The CanCOLD study found that a BEC ≥ 300 cells/μL independently predicts faster lung function decline (7). In non-ICS (inhaled corticosteroids)-treated COPD, people with BEC < 100 cells/μL had more baseline emphysema, prospective exacerbations, and lung function decline (8). Two large 1-year trials of monoclonal antibodies (mepolizumab and benralizumab) that reduced BEC in COPD patients with exacerbation histories showed minimal impact on exacerbation rates, health status, and lung function, indicating that eosinophils may not be a key target for COPD treatment (9, 10).

**Figure 1:**
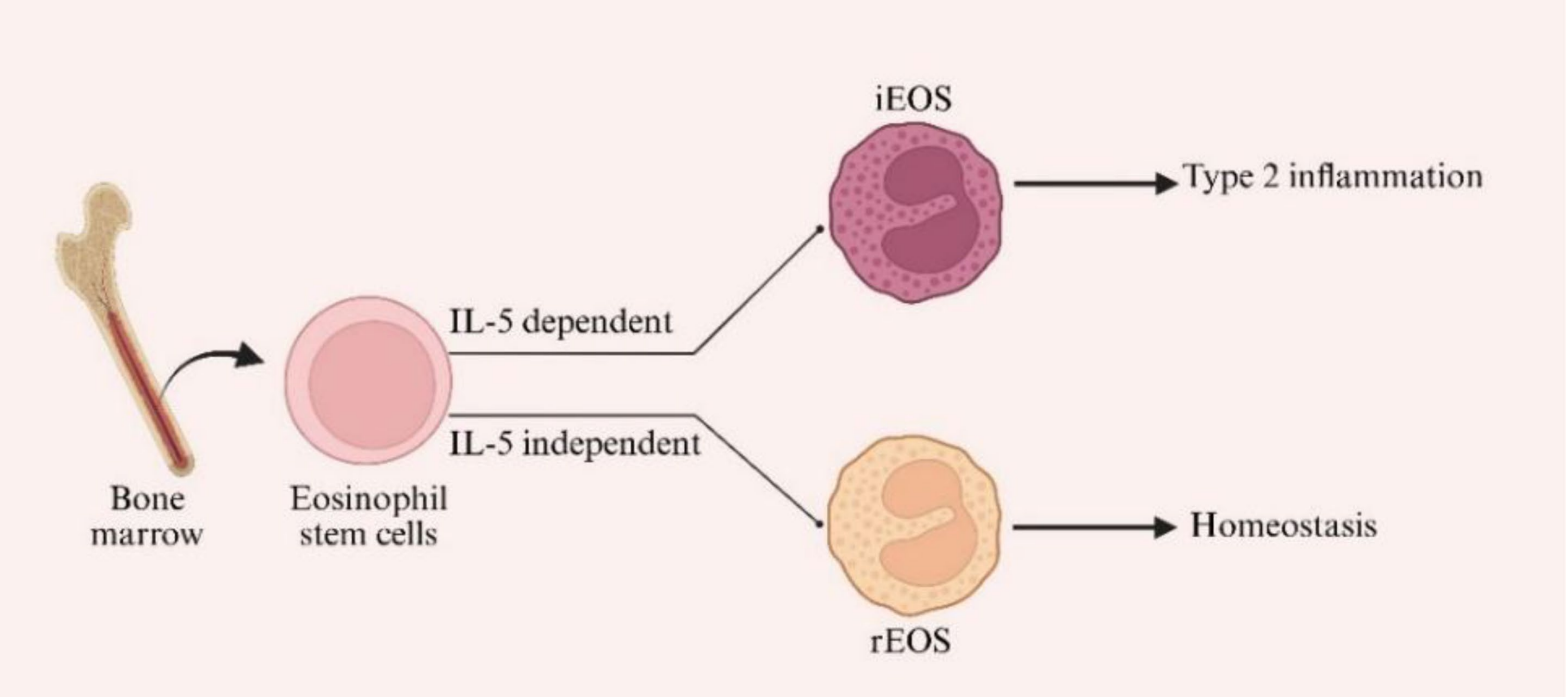
Eosinophilic stem cells are released from the bone marrow, with a subset differentiating into inflammatory eosinophils (iEOS) under the influence of interleukin-5 (IL-5), contributing to the type 2 inflammation. Conversely, another subset remains unaffected by IL-5 and is involved in maintaining homeostasis. iEos, inflammatory eosinophil; rEos, resident eosinophil; IL-5, interleukin-5. Created with BioRender.com.

The concepts of iEos (inflammatory eosinophil) and rEos (resident eosinophil) have been introduced in recent years, and these two cell types exhibit distinct physiological functions (11) (Figure 1). Subtype of iEos which are recruited by IL-5 and appear to be responsible for the abnormal inflammatory response and patients with asthma had a higher proportion of iEos compared with those with COPD (12). On the other hand, rEos is associated with the homeostasis of various systems within the body (13). The variability in research findings regarding the impact of eosinophils on COPD may be attributed to the distinct subtypes of eosinophils.

Currently, there is a paucity of research regarding eosinophil subtypes in patients with COPD. The objective of this study is to compare the eosinophil subtypes in stable COPD patients with those in healthy controls, and to assess their potential role in the pathogenesis of COPD.

## Materials and methods

This study was approved by the ethics committee of Beijing Chao-Yang Hospital and informed consent was obtained from all participants and complies with the Declaration of Helsinki. This analysis comprises data from a total of 27 patients with stable COPD and 10 health control was also included between Jan 2025 and Jun 2025.

The inclusion criteria are listed as follow: age≥40 years old; COPD was defined in accordance with GOLD guidelines as a heterogeneous lung disorder characterized by chronic respiratory symptoms and persistent, often progressive airflow obstruction (post-bronchodilator FEV₁/FVC < 70%), resulting from airway and/or alveolar abnormalities (14); both COPD cohort and the healthy control group consist of individuals who are either current or former smokers; patients with COPD who participated in this study received triple inhaled therapy, and those in a stable condition did not experience any exacerbations for at least 3 months.

Exclusion Criteria: patients exhibiting characteristics of bronchial asthma, positive bronchodilator test and bronchial provocation test; presence of other severe pulmonary diseases (e.g., bronchiectasis); end-stage of chronic disease (e.g., chronic kidney failure, chronic heart failure and malignancy), patients received oral glucocorticoids and immunosuppressors; incomplete clinical data; unwilling to participate.

Baseline characteristics (gender, age, smoking history, comorbidities) were systematically recorded, with the control cohort matched to the COPD group by these variables to ensure comparability. Pulmonary function tests (FEV₁%, FEV₁/FVC) were performed in both groups. C-reactive protein (CRP), routine hematological parameters, and neutrophil-to-lymphocyte ratio (NLR) were assessed.

### Blood eosinophil subtype by flow cytometry analysis

The eosinophils were labeled according to the Mesnil criteria as rEos (Siglec-8^+^CD62L^+^IL-3R^lo^)or iEos (Siglec-8^+^CD62L^lo^IL-3R^hi^) (13).100 μL of whole blood was transferred into a flow cytometry tube, followed by the addition of red blood cell (RBC) lysis buffer. To stain surface antigens, premixed surface antibody solution was added. This solution was made by PE-conjugated anti-human Siglec-8, PE/Dazzle™ 594-conjugated anti-human CD62L, and BV510-conjugated mouse anti-human IL-3Rα (CD123). The mixture was incubated for 30 min at room temperature in the dark, then centrifuged at 500 × g for 5 min to remove unbound antibodies. Single-color compensation beads were used to adjust fluorescence compensation, and all samples were analyzed on a flow cytometer. Data acquisition and analysis were performed using dedicated flow cytometry software. Fluorescence-minus-one (FMO) controls were systematically included for each staining panel—prepared by omitting one fluorochrome-conjugated antibody at a time while retaining all other components—to set accurate gating thresholds for Siglec-8, CD62L, and CD123, distinguishing specific fluorescence from background noise and minimizing false-positive signals. The flow cytometry detection method utilized in this study is based on the methodology outlined in the Mesnil’s study (13).

The enzyme-linked immunosorbent assay (ELISA) was used to quantify cytokine levels. Serum was isolated from peripheral blood samples following standard protocols, and cytokines were directly measured using validated ELISA kits, including interleukin (IL)-4, IL-5, IL-13, major basic protein (MBP), C-C motif chemokine ligand (CCL) 11, CCL24, and eosinophil cationic protein (ECP).

Currently, the literature lacks adequate information to precisely ascertain the required sample size for this investigation. Consequently, conducting a pilot study is imperative to examine the influence of eosinophil subtypes on the incidence of COPD.

### Statistical analysis

Data were presented as mean±standard deviation (normal distribution) or median (interquartile range, IQR) (non-normal distribution). Differences in smoking status and comorbidities across study groups were compared using Fisher’s exact test (two-sided). Other categorical variables were compared using the Chi-square test. Continuous variables were tested for normality via the Kolmogorov–Smirnov test; normally distributed data were analyzed with Student’s *t*-test, and non-normally distributed data with the Mann–Whitney *U*-test. Receiver-operating characteristic (ROC) curves were constructed to evaluate markers’ predictive performance for COPD presence. Logistic regression with conditional forward stepwise selection was used to identify markers independently associated with COPD, reporting adjusted odds ratios (ORs, 95% CIs). A prediction model was constructed using a classification and regression tree (CRT), and the robustness of the model and the risk of overfitting were evaluated through 5-fold cross validation. The binary logistic regression model’s performance was thoroughly assessed using the C-statistic (AUC) for discrimination and the Hosmer-Lemeshow test and calibration curve for calibration accuracy. Analyses were two-tailed, with significance set at *p* < 0.05.

## Results

Eosinophilic progenitor cells are mobilized from the bone marrow, with a specific subset differentiating into inflammatory eosinophils (iEos) under the influence of interleukin-5 (IL-5), thereby contributing to type 2 inflammatory responses. In contrast, another subset (resident eosinophil, rEos) remains unresponsive to IL-5 and plays a role in maintaining homeostatic balance.

There were no statistically significant differences in terms of age, gender, smoking status and comorbidity (including hypertension, coronary heart disease and diabetes) between the patients with stable COPD and the control group. The hemoglobin levels in patients with COPD were significantly elevated compared to those in the control group. When comparing patients with stable COPD to the control group, there were no statistically significant differences in CRP, leukocyte count, neutrophil count, lymphocyte count, NLR, eosinophil count, or platelet count. There were significant differences in terms of FEV_1_/FVC and FEV_1_% between stable COPD and healthy controls (Table 1).

**Table 1:** Comparison between stable COPD and control.

Characteristics	Stable COPD (*n* = 27)	Controls (*n* = 10)	*p*
Age	64 ± 9	66 ± 10	0.554
Gender (male, %)	24 (88.9%)	7 (70%)	0.313
Smoking status (current, %)	19 (70.4%)	4 (40%)	0.132
Smoking status (former, %)	8 (29.6%)	6 (60%)	
Hypertension (*n*, %)	8 (29.6%)	2 (20%)	0.694
Coronary heart disease (*n*, %)	8 (29.6%)	3 (30%)	1
Diabetes (*n*, %)	4 (14.8%)	2 (20%)	0.653
CRP (mg/L)	4.7 (1.1–9)	3.2 (2–5.4)	0.827
Leukocyte count (x10^9^/L)	7.12 ± 1.76	6.55 ± 1.91	0.397
Neutrophil count (x10^9^/L)	4.70 ± 1.60	4.05 ± 1.74	0.29
Lymphocytes count (x10^9^/L)	1.84 ± 0.61	1.95 ± 0.83	0.661
NLR	2.16 (1.73–3.55)	1.74 (1.36–2.32)	0.182
Hemoglobin (g/L)	146 ± 13	134 ± 18	0.028
Platelet count (x10^9^/L)	215 (194–256)	214 (200–237)	0.933
Eosinophils count (x10^9^/L)	0.19 ± 0.13	0.11 ± 0.04	0.081
FEV_1_/FVC	60 (42–64)	83.5 (79–85)	<0.001
FEV_1_%	68 (50–86)	86 (82–87)	0.028
Mild (FEV_1_% ≥ 80%)	7 (25.9%)	—	—
Moderate (50% ≤ FEV_1_ < 80%)	8 (27.6%)	—	—
Severe (30% ≤ FEV_1_% < 50%)	10 (37%)	—	—
Very severe (FEV_1_% < 30%)	2 (7.4%)	—	—

COPD, chronic obstructive pulmonary disease; CRP, C-reactive protein; NRL, neutrophil/lymphocyte ratio; FEV_1_, Forced expiratory volume in 1 s; FVC, Forced vital capacity.

Smoking status, hypertension, coronary heart disease and diabetes were compared using Fisher’s exact test (two-sided).

There were no significant differences observed in the total number of eosinophils, as well as in the total number of inflammatory and resident eosinophils, between patients with stable COPD and healthy controls. Additionally, no significant differences were detected in the absolute number of iEos or in the proportion of iEos relative to the total eosinophil count. However, the proportion of eosinophils in the patient group was significantly higher than in the control group. Furthermore, both the absolute number of rEos and the proportion of rEos relative to the total eosinophil count were significantly decreased in patients with COPD compared to controls. No significant differences were identified concerning cytokines (including IL-4, IL-5, IL-13, MBP, CCL11, CCL24 and ECP) between these two groups (Table 2).

**Table 2:** Results of flow cytometry and cytokines.

Characteristics	Stable COPD (*n* = 27)	Controls (*n* = 10)	*p*
Total number of eosinophils	2,286 (1814–2,950)	1934 (1567–2,252)	0.072
Proportion of eosinophils (%)	2.14 (1.67–2.83)	1.57 (1.35–1.71)	0.006
Total number of iEos	1,023 (623–1,356)	720 (640–865)	0.171
Total number of rEos	95 (49–136)	115 (98–172)	0.166
The absolute number of iEos (cell/uL)	13.64 (9.02–20.83)	17.16 (15.28–22.78)	0.151
Proportion of iEos to total eosinophils (%)	41.01 (35.63–47.06)	39.42 (34.63–45.23)	0.682
The absolute number of rEos (cell/uL)	1.15 (0.72–2.15)	2.79 (2.16–3.68)	0.001
Proportion of rEos to total eosinophils (%)	3.38 (2.14–5.31)	6.88 (5.35–7.96)	0.003
IL-4	0.45 (0.37–0.69)	0.41 (0.29–0.42)	0.115
IL-5	1.87 (1.51–5.4)	1.72 (1.04–2.23)	0.194
IL-13	6.61 ± 7.93	19.976 ± 41.56	0.112
MBP	0.13 ± 0.26	0.11 ± 0.11	0.723
CCL11	19.02 (7.64–36.60)	24.49 (14.67–32)	0.694
CCL24	618.91 ± 608	372.72 ± 201.66	0.221
ECP	26.83 (17.11–41.13)	21.69 (11.39–54.29)	0.392

COPD, chronic obstructive pulmonary disease; iEos, inflammatory eosinophil; rEos, resident eosinophil; IL-4, interleukin-4; IL-5, interleukin-5; IL-13, interleukin-13; MBP, major basic protein; CCL11, C-C motif chemokine ligand 11; CCL24, C-C motif chemokine ligand 24; ECP, eosinophil cationic protein.

**Figure 2:**
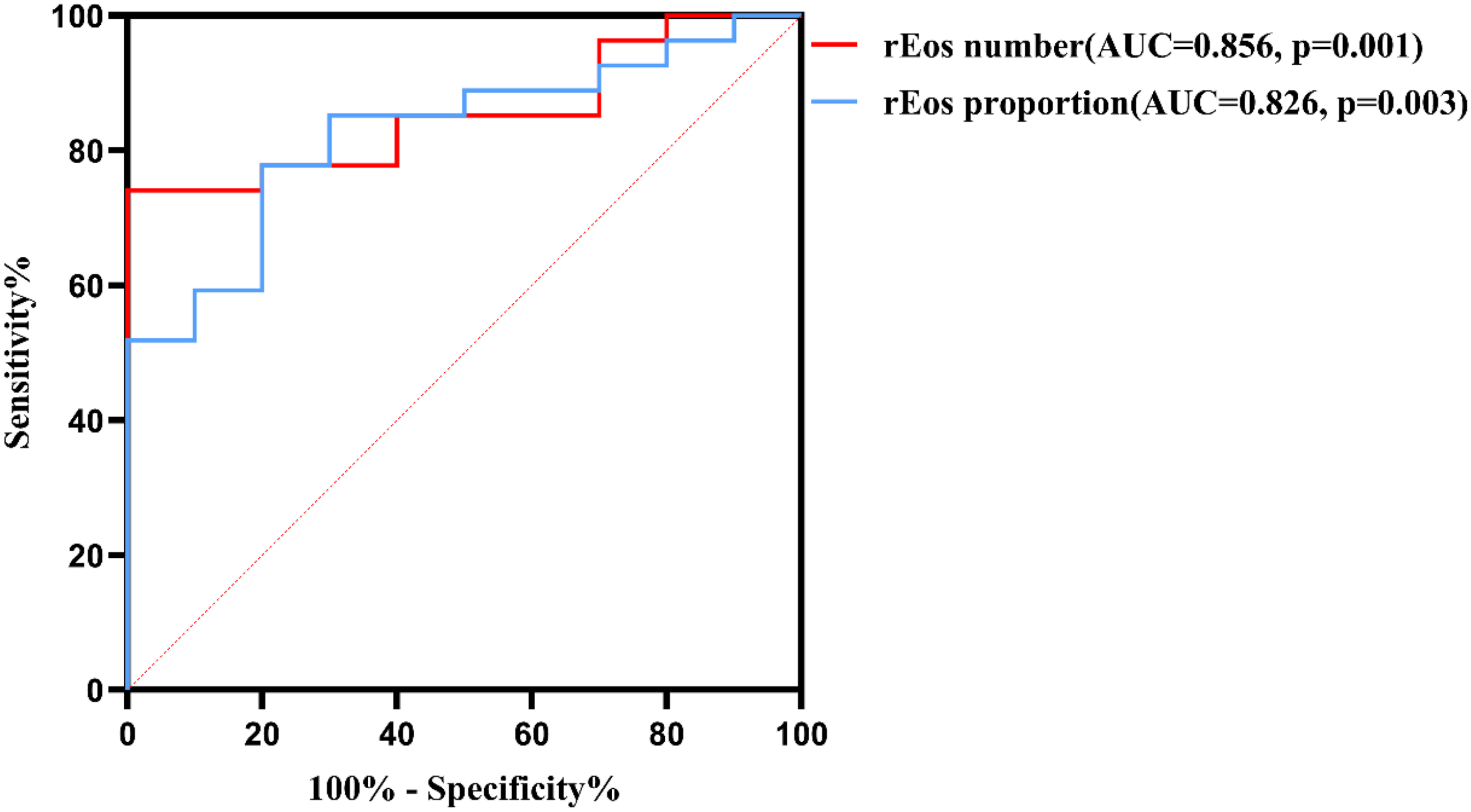
Receiver-operating characteristic curves of the absolute number of rEos and proportion of rEos to total eosinophils for predicting COPD. iEos, inflammatory eosinophil; rEos, resident eosinophil; AUC, area under the curve.

The ROC curves of the absolute number of rEos and proportion of rEos to total eosinophils were displayed in Figure 2, Table 3 showed that the AUC of the absolute number of rEos (0.856; 95% CI, 0.736–0.975) was higher than that of proportion of rEos to total eosinophils (0.826; 95% CI, 0.690–0.962).

**Table 3:** ROC curve data.

Characteristics	The absolute number of rEos (cell/uL)	Proportion of rEos to total eosinophils (%)
Cutoff value	<1.955	<0.053
Sensitivity, %	0.741	0.778
Specificity, %	1	0.8
Positive predictive value, %	1	0.913
Negative predictive value, %	0.588	0.571
Diagnostic accuracy	0.811	0.784
Youden’s index	0.741	0.578
AUC	0.856	0.826
95%CI	0.736–0.975	0.690–0.962
*p* value	0.001	0.003

rEos, resident eosinophil; AUC, area under the curve; CI, confidence interval.

The diagnostic accuracy of the absolute number of rEos and proportion of rEos to total eosinophils were 0.811 and 0.784. The cutoff value for predicting COPD was the absolute number of rEos<1.955 and proportion of rEos to total eosinophils <0.053.

In the logistic regression analysis, age, gender, smoking status, leukocyte count, NLR, hemoglobin, and the absolute number of rEos were included as variables. No significant correlation was observed between these variables and the total eosinophil count (see Supplementary Table S1). The analysis revealed that the absolute number of rEos is independently associated with COPD presence (Table 4).

**Table 4:** Logistic regression analysis of risk factors for COPD.

Characteristics	B	S.E.	Wals	df	Sig	OR (95% CI)
Age	0.012	0.076	0.025	1	0.873	1.012 (0.872–1.175)
Gender	1.141	1.566	0.531	1	0.466	3.13 (0.145–67.421)
Smoking status	−0.102	1.3	0.006	1	0.938	0.903 (0.071–11.537)
Leukocyte count	0.355	0.342	1.079	1	0.299	1.426 (0.73–2.788)
NLR	0.065	0.197	0.109	1	0.741	1.067 (0.726–1.57)
Hemoglobin	0.077	0.048	2.632	1	0.105	1.08 (0.984–1.186)
The absolute number of rEos (cell/uL)	−1.465	0.659	4.941	1	0.026	0.n (0.063–0.841)

rEos, resident eosinophil; AUC, area under the curve; NRL, neutrophil/lymphocyte ratio; OR, odds ratio; CI, confidence interval.

A Classification and Regression Tree (CRT) model predicting COPD was developed using age, gender, smoking status, leukocyte count, NLR, hemoglobin, and absolute rEos count as predictors. Five-fold cross-validation confirmed no significant overfitting (resubstitution error = 0.108, SE = 0.051; cross-validation error = 0.324, SE = 0.077), supporting the model’s generalizability (see Supplementary Table S2).

The binary logistic regression model for COPD presence prediction showed statistical reliability: excellent discriminative ability (training-set AUC = 0.919, 95% CI: 0.829–1.000; cross-validated AUC = 0.78, 95% CI: 0.67–0.89), satisfactory calibration (Hosmer-Lemeshow test: *χ^2^* = 3.742, df = 7, *p* = 0.809), 83.8% overall accuracy, and no overfitting (5-fold cross-validation) (see Supplementary Table S3).

## Discussion

Investigating the subtypes of eosinophils and their differential involvement in the pathogenesis of COPD may yield critical insights for enhancing disease management strategies. Our study demonstrated that patients with COPD had much lower level of rEos than health controls and absolute number of rEos was the independently associated with the occurrence of COPD.

Numerous studies have demonstrated that blood eosinophil counts can predict the magnitude of ICS in preventing future exacerbations and consequently, the Global Initiative for Chronic Obstructive Lung Disease (GOLD) recommends utilizing BEC to guide the use of ICS within pharmacological management strategies (15, 16). Evidence suggests that, on average, BEC are elevated in patients with COPD, despite a significant overlap with control groups (17, 18). The repeatability of BEC in a large primary care population is generally reasonable (19); however, increased variability is noted at elevated thresholds (20) and improved reproducibility is observed at lower thresholds (21). Elevated BEC and FeNO (fractional exhaled nitric oxide) is associated with accelerated FEV_1_ decline in individuals with chronic airway disease in the general population (22). However, several studies have yielded varying outcomes concerning the predictive capacity of blood eosinophils for future exacerbation events, with findings indicating either no correlation (23) or a positive association (24).

Research conducted by Mesnil demonstrated the presence of two distinct types of eosinophils within solid tissue in a murine model and revealed difference in the eosinophils’ localization, nuclear morphology, and surface protein expression. Mesnil et al. (13): one IL-5 dependent leading to inflammatory cells and one IL-5 independent, leading to resident cells. Under normal conditions, the bone marrow releases few eosinophils, but their production significantly increases due to Th2 cell responses during helminth infections or allergic diseases like asthma (25, 26). The Th2-associated cytokine IL-5 is intricately linked to the eosinophil lineage, playing a pivotal role in the proliferation of eosinophils from committed progenitors in the bone marrow. Additionally, IL-5 is essential for the mobilization of these cells into the bloodstream and for their subsequent survival following tissue migration (27). On the other hand, rEos as opposed to iEos, do not depend on IL-5 for their presence in the blood or recruitment to the lungs. Actually, Tissue-resident eosinophils (rEos) are predominantly located in the gastrointestinal tract, adipose tissue, thymus, uterus, and mammary glands, where they play a crucial role in regulating a range of essential biological functions (28). Tissue-resident eosinophils in the gastrointestinal tract aid IgA class switching, maintain IgA plasma cells, and support Peyer’s patches and mucus production (29). In adipose tissue, they produce IL-4, promoting alternatively activated macrophages (30). In the thymus, rEos may induce thymocyte apoptosis, contributing to negative T cell selection (31). They are also suggested to prepare the uterus for pregnancy and regulate mammary gland development (32). Mesnil also illustrated that rEos are not affected by allergic inflammation and are independent of IL-5 for their presence in the lung, and rEos can inhibit the development of Th2 responses to house dust mite and the pro-Th2 potential of dendritic cells. They also found similar numbers of eosinophils in the lung tissue of both healthy and asthmatic individuals, indicating stable levels of parenchymal eosinophils regardless of inflammation. In asthmatic lungs, additional iEos eosinophils were present in the peribronchial area, likely representing infiltrating eosinophils in the bronchi (13).

Our study found no difference in blood eosinophil levels between stable COPD patients and controls in routine blood test. However, flow cytometry revealed significantly lower rEos number and proportion in patients with COPD, additionally, rEos is be linked to COPD pathophysiology in the regression analysis. The findings of this study align with those of Mesnil, indicating that rEos may exerts an inhibitory effect on the onset of airway inflammation and elevated levels of rEos may confer a protective effect within the respiratory system.

In the ROC curve analysis, the area under the curve (AUC) for the absolute number of the rEos is the largest, indicating the highest sensitivity and specificity. In regression analysis, rEos values below 1.955 are significant predictors of COPD occurrence. This finding serves as an important consideration for clinicians. When screening for COPD in high-risk populations, rEos levels should be considered and clinicians should remain vigilant for the development of COPD in populations exhibiting low rEos levels.

Previous studies indicate that asthma patients have higher iEos levels than those with COPD (12). Mepolizumab treatment alters iEos levels, but not rEos (33). The role of eosinophil subtypes in COPD remains unclear. Previous studies suggest iEos needs IL-5 activation, and since IL-5 levels are similar in both groups, this might explain the lack of difference in iEos. Larger studies including COPD patients with acute exacerbations might uncover iEos differences.

There was no statistically significant difference in IL-5 levels between patients with stable COPD and the control group, nor in the absolute number and proportion of iEos. Our study indicates that the inflammatory eosinophil response mediated by IL-5 may not be significant in patients with stable COPD who have an eosinophil count of 0.19 ± 0.13. Our findings are consistent with prior research, indicating that mepolizumab, as compared with placebo, on the annual rate of moderate or severe exacerbations was found among patients with higher blood eosinophil counts (>300 cells/uL) at screening (9). It is indeed that eosinophil counts fluctuate in stable COPD patients, with greater variations in those with high counts (34), and ICS exert a specific regulatory effect on blood eosinophil levels in patients with COPD (35). All patients enrolled in this study received triple inhaled therapy for at least 3 months. While our study does not address the impact of ICS on eosinophil subsets, the consistent baseline ICS exposure among all participants ensures that our findings reflect the profile of eosinophil subsets in stable COPD patients receiving triple inhaled therapy. This standardized baseline allows for reliable comparisons of eosinophil subset distributions between stable COPD patients and controls, and provides foundational data for future studies investigating the regulatory effects of ICS on eosinophil subtyping in COPD.

Eosinophil-associated cytokines constitute a crucial regulatory network in the pathogenesis of the eosinophilic phenotype of COPD. IL-4 and IL-13 contribute to airway remodeling, while chemokines CCL11 and CCL24 facilitate the specific recruitment of eosinophils to the airway, and MBP and ECP are implicated in inducing epithelial damage (36). However, our study did not reveal a statistically significant difference between the stable COPD group and the healthy control group. This outcome is likely attributable to the limited sample size and the fact that the COPD patients included in the study were in a stable phase of the disease.

It has been acknowledged that eosinophils are recruited to the lungs, where they secrete a variety of chemokines, cytokines, and cytotoxic granular products, thereby contributing to inflammation among patients with COPD (37). This study exclusively examined the phenotype of peripheral blood eosinophils, without assessing eosinophils present in airway and lung tissues. Nonetheless, prior research has indicated a potential correlation between peripheral blood eosinophils and those in the airways (38). Building on the findings of this study, we intend to undertake fundamental experiments in the future to compare the phenotypic characteristics of eosinophils in the airways and lungs of patients with COPD.

Prior studies demonstrated that alterations associated with eosinophilic inflammation, such as airway remodeling, mucus plugging, and immune response dysregulation, are likely significant contributors to the deterioration of health and exacerbations in patients with COPD (39, 40). IL-5 is a pivotal type 2 inflammatory cytokine that facilitates the differentiation, proliferation, maturation, migration, and survival of eosinophils within the bloodstream and tissues (41). The role of iEos in asthma is well-established; however, the characterization of eosinophil subtypes in individuals with COPD remains insufficiently understood. Our study indicates that a reduction in rEos may be independently associated with the onset of COPD, and a decrease in rEos may enhance the pulmonary inflammatory response to stimuli, potentially resulting in an exacerbation of inflammation.

## Conclusion

rEos was significantly reduced in the stable COPD cohort compared to the healthy controls, and was associated with the onset of COPD. Future research should validate the role of rEos in COPD pathogenesis via larger, more diverse cohorts in well-designed multicenter prospective studies. Additionally, fundamental experiments are needed to elucidate the molecular and cellular mechanisms underlying eosinophilic phenotypes in COPD.

### Limitation

This study had certain limitations. Firstly, this cross-sectional study offers a single-time snapshot, preventing the establishment of causal relationships and leaving room for reverse causality and residual confounding. Its design also limits the observation of temporal changes and long-term outcomes, and may introduce recall or selection bias. Secondly, this study was conducted at a single center and involved a relatively small sample size, highlighting the need for future multi-center replication studies. Other outcomes, including long-term symptom control and mortality, remain insufficiently clarified due to small sample sizes and limited follow-up periods. Thirdly, the present study did not establish a direct correlation between rEos and the inhibition of airway inflammation, necessitating further research to confirm these findings. Lastly, individual samples were not analyzed in duplicate due to the limited sample size of this pilot investigation. In future large-scale multicenter studies with an expanded cohort to strengthen the robustness of findings related to eosinophil subset quantification.

## Data Availability

The raw data supporting the conclusions of this article will be made available by the authors, without undue reservation.

## References

[1] O'Donnell R Breen D Wilson S Djukanovic R. Inflammatory cells in the airways in COPD. Thorax. (2006) 61:448–54. doi: 10.1136/thx.2004.024463, 16648353 PMC2111192

[2] Saha S Brightling CE. Eosinophilic airway inflammation in COPD. Int J Chron Obstruct Pulmon Dis. (2006) 1:39–47. doi: 10.2147/copd.2006.1.1.39, 18046901 PMC2706606

[3] Siva R Green RH Brightling CE Shelley M Hargadon B McKenna S . Eosinophilic airway inflammation and exacerbations of COPD: a randomised controlled trial. Eur Respir J. (2007) 29:906–13. doi: 10.1183/09031936.00146306 17301099

[4] Brightling CE Monteiro W Ward R Parker D Morgan MD Wardlaw AJ . Sputum eosinophilia and short-term response to prednisolone in chronic obstructive pulmonary disease: a randomised controlled trial. Lancet. (2000) 356:1480–5. doi: 10.1016/S0140-6736(00)02872-5 11081531

[5] Couillard S Larivee P Courteau J Vanasse A. Eosinophils in COPD exacerbations are associated with increased readmissions. Chest. (2017) 151:366–73. doi: 10.1016/j.chest.2016.10.003, 27746201

[6] Greulich T Tuffers J Mager S Eder A Maxheim M Alter P . High eosinophil blood counts are associated with a shorter length of hospital stay in exacerbated COPD patients—a retrospective analysis. Respir Res. (2020) 21:106. doi: 10.1186/s12931-020-01365-5, 32375777 PMC7204070

[7] Tan WC Bourbeau J Nadeau G Wang W Barnes N Landis SH . High eosinophil counts predict decline in FEV(1): results from the CanCOLD study. Eur Respir J. (2021) 57:2000838. doi: 10.1183/13993003.00838-2020 33303555

[8] LeMaster WB Quibrera PM Couper D Tashkin DP Bleecker ER Doerschuk CM . Clinical implications of low absolute blood eosinophil count in the SPIROMICS COPD cohort. Chest. (2023) 163:515–28. doi: 10.1016/j.chest.2022.10.029, 36343688 PMC10083128

[9] Pavord ID Chanez P Criner GJ Kerstjens HAM Korn S Lugogo N . Mepolizumab for eosinophilic chronic obstructive pulmonary disease. N Engl J Med. (2017) 377:1613–29. doi: 10.1056/nejmoa1708208, 28893134

[10] Criner GJ Celli BR Brightling CE Agusti A Papi A Singh D . Benralizumab for the prevention of COPD exacerbations. N Engl J Med. (2019) 381:1023–34. doi: 10.1056/NEJMoa1905248, 31112385

[11] Januskevicius A Jurkeviciute E Janulaityte I Kalinauskaite-Zukauske V Miliauskas S Malakauskas K. Blood eosinophils subtypes and their survivability in asthma patients. Cells. (2020) 9:1248. doi: 10.3390/cells9051248, 32443594 PMC7291159

[12] Cabrera Lopez C Sanchez Santos A Lemes Castellano A Cazorla Rivero S Brena Atienza J Gonzalez Davila E . Eosinophil subtypes in adults with asthma and adults with chronic obstructive pulmonary disease. Am J Respir Crit Care Med. (2023) 208:155–62. doi: 10.1164/rccm.202301-0149OC, 37071848

[13] Mesnil C Raulier S Paulissen G Xiao X Birrell MA Pirottin D . Lung-resident eosinophils represent a distinct regulatory eosinophil subset. J Clin Invest. (2016) 126:3279–95. doi: 10.1172/JCI85664, 27548519 PMC5004964

[14] Agusti A Celli BR Criner GJ Halpin D Anzueto A Barnes P . Global initiative for chronic obstructive lung disease 2023 report: GOLD executive summary. Eur Respir J. (2023) 61:2300239. doi: 10.1183/13993003.00239-2023, 36858443 PMC10066569

[15] Bafadhel M Peterson S De Blas MA Calverley PM Rennard SI Richter K . Predictors of exacerbation risk and response to budesonide in patients with chronic obstructive pulmonary disease: a post-hoc analysis of three randomised trials. Lancet Respir Med. (2018) 6:117–26. doi: 10.1016/S2213-2600(18)30006-7, 29331313

[16] Lipson DA Barnhart F Brealey N Brooks J Criner GJ Day NC . Once-daily single-inhaler triple versus dual therapy in patients with COPD. N Engl J Med. (2018) 378:1671–80. doi: 10.1056/nejmoa1713901, 29668352

[17] Hartl S Breyer MK Burghuber OC Ofenheimer A Schrott A Urban MH . Blood eosinophil count in the general population: typical values and potential confounders. Eur Respir J. (2020) 55:1901874. doi: 10.1183/13993003.01874-2019, 32060069

[18] Kolsum U Southworth T Jackson N Singh D. Blood eosinophil counts in COPD patients compared to controls. Eur Respir J. (2019) 54:1900633. doi: 10.1183/13993003.00633-2019, 31221811

[19] Landis SH Suruki R Hilton E Compton C Galwey NW. Stability of blood eosinophil count in patients with COPD in the UK clinical practice research datalink. COPD. (2017) 14:382–8. doi: 10.1080/15412555.2017.1313827, 28569614

[20] Oshagbemi OA Burden AM Braeken DCW Henskens Y Wouters EFM Driessen JHM . Stability of blood eosinophils in patients with chronic obstructive pulmonary disease and in control subjects, and the impact of sex, age, smoking, and baseline counts. Am J Respir Crit Care Med. (2017) 195:1402–4. doi: 10.1164/rccm.201701-0009le, 28165763

[21] Southworth T Beech G Foden P Kolsum U Singh D. The reproducibility of COPD blood eosinophil counts. Eur Respir J. (2018) 52:1800427. doi: 10.1183/13993003.00427-2018, 29724922

[22] Colak Y Afzal S Marott JL Vestbo J Nordestgaard BG Lange P. Type-2 inflammation and lung function decline in chronic airway disease in the general population. Thorax. (2024) 79:349–58. doi: 10.1136/thorax-2023-220972, 38195642 PMC10958305

[23] Casanova C Celli BR de-Torres JP Martinez-Gonzalez C Cosio BG Pinto-Plata V . Prevalence of persistent blood eosinophilia: relation to outcomes in patients with COPD. Eur Respir J. (2017) 50:1701162. doi: 10.1183/13993003.01162-2017, 29167301

[24] Yun JH Lamb A Chase R Singh D Parker MM Saferali A . Blood eosinophil count thresholds and exacerbations in patients with chronic obstructive pulmonary disease. J Allergy Clin Immunol. (2018) 141:2037–47. doi: 10.1016/j.jaci.2018.04.010 29709670 PMC5994197

[25] Rosenberg HF Dyer KD Foster PS. Eosinophils: changing perspectives in health and disease. Nat Rev Immunol. (2013) 13:9–22. doi: 10.1038/nri3341, 23154224 PMC4357492

[26] Travers J Rothenberg ME. Eosinophils in mucosal immune responses. Mucosal Immunol. (2015) 8:464–75. doi: 10.1038/mi.2015.2, 25807184 PMC4476057

[27] Rothenberg ME Hogan SP. The eosinophil. Annu Rev Immunol. (2006) 24:147–74. doi: 10.1146/annurev.immunol.24.021605.090720 16551246

[28] Lee JJ Jacobsen EA McGarry MP Schleimer RP Lee NA. Eosinophils in health and disease: the LIAR hypothesis. Clin Exp Allergy. (2010) 40:563–75. doi: 10.1111/j.1365-2222.2010.03484.x, 20447076 PMC2951476

[29] Jung Y Wen T Mingler MK Caldwell JM Wang YH Chaplin DD . IL-1beta in eosinophil-mediated small intestinal homeostasis and IgA production. Mucosal Immunol. (2015) 8:930–42. doi: 10.1038/mi.2014.123 25563499 PMC4481137

[30] Wu D Molofsky AB Liang HE Ricardo-Gonzalez RR Jouihan HA Bando JK . Eosinophils sustain adipose alternatively activated macrophages associated with glucose homeostasis. Science. (2011) 332:243–7. doi: 10.1126/science.1201475, 21436399 PMC3144160

[31] Throsby M Herbelin A Pleau JM Dardenne M. CD11c+ eosinophils in the murine thymus: developmental regulation and recruitment upon MHC class I-restricted thymocyte deletion. J Immunol. (2000) 165:1965–75. doi: 10.4049/jimmunol.165.4.1965, 10925279

[32] Gouon-Evans V Pollard JW. Eotaxin is required for eosinophil homing into the stroma of the pubertal and cycling uterus. Endocrinology. (2001) 142:4515–21. doi: 10.1210/endo.142.10.8459, 11564717

[33] Miguens-Suarez P Martelo-Vidal L Vazquez-Mera S Blanco-Aparicio M Calvo-Alvarez U Gonzalez-Fernandez C . Mepolizumab treatment alters the functional phenotype of eosinophils in severe eosinophilic asthma. Front Immunol. (2025) 16:1654111. doi: 10.3389/fimmu.2025.1654111, 41394834 PMC12695602

[34] Baraldi F Bartlett-Pestell S Allinson JP Macleod M Mah J Bloom C . Blood eosinophil count stability in chronic obstructive pulmonary disease and the eosinophilic exacerbator phenotype. Am J Respir Crit Care Med. (2025) 211:870–2. doi: 10.1164/rccm.202407-1287RL, 39813681 PMC12091026

[35] Baloira Villar A Pallares Sanmartin A Nunez Fernandez M. Eosinophils and inhaled corticosteroids in chronic obstructive pulmonary disease. Arch Bronconeumol. (2016) 52:541. doi: 10.1016/j.arbr.2016.07.026, 27444238

[36] Lee YL Heriyanto DS Yuliani FS Laiman V Choridah L Lee KY . Eosinophilic inflammation: a key player in COPD pathogenesis and progression. Ann Med. (2024) 56:2408466. doi: 10.1080/07853890.2024.2408466, 39624959 PMC11459840

[37] David B Bafadhel M Koenderman L De Soyza A. Eosinophilic inflammation in COPD: from an inflammatory marker to a treatable trait. Thorax. (2021) 76:188–95. doi: 10.1136/thoraxjnl-2020-215167, 33122447 PMC7815887

[38] Zhang H Lian M Dilixiati N Song J Yang J Lin R . The value of FeNO and peripheral blood eosinophils in assessing airway eosinophilic inflammation in patients with acute exacerbations of chronic obstructive pulmonary disease. Cytopathology. (2026) 37:160–6. doi: 10.1111/cyt.70009, 40751386

[39] Vedel-Krogh S Nielsen SF Lange P Vestbo J Nordestgaard BG. Blood eosinophils and exacerbations in chronic obstructive pulmonary disease. The Copenhagen general population study. Am J Respir Crit Care Med. (2016) 193:965–74. doi: 10.1164/rccm.201509-1869OC, 26641631

[40] Singh D Agusti A Martinez FJ Papi A Pavord ID Wedzicha JA . Blood eosinophils and chronic obstructive pulmonary disease: a global initiative for chronic obstructive lung disease science committee 2022 review. Am J Respir Crit Care Med. (2022) 206:17–24. doi: 10.1164/rccm.202201-0209PP, 35737975

[41] Bajbouj K AbuJabal R Sahnoon L Olivenstein R Mahboub B Hamid Q. IL-5 receptor expression in lung fibroblasts: potential role in airway remodeling in asthma. Allergy. (2023) 78:882–5. doi: 10.1111/all.15627, 36575907

