# Supplementary material for: Resident eosinophils in patients with chronic obstructive pulmonary disease: a pilot study
Source: Front Med (Lausanne). 2026 May 25;13:1814890. doi: 10.3389/fmed.2026.1814890 (PMC13243407; doi:10.3389/fmed.2026.1814890)
Supplement: Supplementary file 1 [file Data_Sheet_1.PDF]

Table 1 Analysis of the correlation between independent variables in the regression and the total eosinophil count

	r	*P*
Age	-0.002	0.991
Gender	-0.086	0.613
Smoking status	-0.013	0.939
Leukocyte count	0.229	0.173
NLR	0.067	0.693
Hemoglobin	-0.081	0.632
The absolute number of rEos (cell/uL)	0.138	0.417

Abbreviations: NLR= neutrophil/lymphocyte ratio

Table 2 Comparison of Prediction Errors in the CRT Model via 5-Fold Cross-Validation

Evaluation Method	Error Estimate	Standard Error (SE)
Resubstitution (Training Set)	0.108	0.051
5-Fold Cross-Validation	0.324	0.077

Abbreviations: CRT= Classification and Regression Tree

The CRT (Classification and Regression Tree) algorithm developed a predictive model for COPD, using age, gender, smoking status, leukocyte count, NLR, hemoglobin and the absolute number of rEos as predictors. COPD was the dependent variable. To prevent overfitting and enhance generalizability, 5-fold cross-validation was applied. Detailed results follow:

Table 2 shows the model's training set error was 0.108 (SE = 0.051), indicating 89.2% accuracy. After 5-fold cross-validation, the error increased to 0.324 (SE = 0.077), and this difference indicates that the resubstitution error may carry inherent optimism due to the model’s familiarity with the training data. However, the cross-validation effectively corrected this bias, and the absence of an extreme discrepancy between the two error estimates suggests no significant overfitting.

Table 3 Logistic Regression Model Performance and Reliability Metrics

Assessment Dimension	Metric	Value
Discriminative Ability	Training-set C-statistic (AUC)	0.919 (95% CI: 0.829–1.000)
	Cross-validated C-statistic (AUC)	0.78 (95% CI: 0.67–0.89)
	Overall Classification Accuracy	83.8%
Calibration	Hosmer-Lemeshow Test χ^2	3.742
	Degrees of Freedom (df)	7
	Hosmer-Lemeshow Test P-value	0.809
Overfitting Control	Validation Method	5-fold Cross-validation

The binary logistic regression model demonstrates statistical reliability in predicting the presence of COPD, as evidenced by its excellent discriminative capacity (training-set AUC = 0.919, 95% CI: 0.829–1.000; cross-validated AUC = 0.78), satisfactory calibration (Hosmer-Lemeshow test: χ^2=3.742, df=7, P=0.809), and an overall classification accuracy of 83.8%. These findings substantiate the model's robustness and clinical applicability, indicating no signs of overfitting.
